# Reactive Oxygen Species and Inhibitors of Inflammatory Enzymes, NADPH Oxidase, and iNOS in Experimental Models of Parkinson's Disease

**DOI:** 10.1155/2012/823902

**Published:** 2012-04-22

**Authors:** Sushruta Koppula, Hemant Kumar, In Su Kim, Dong-Kug Choi

**Affiliations:** Department of Biotechnology, Research Institute of Inflammatory Diseases, Konkuk University, Chungju 380-701, Republic of Korea

## Abstract

Reactive oxygen species (ROSs) are emerging as important players in the etiology of neurodegenerative disorders including Parkinson's disease (PD). Out of several ROS-generating systems, the inflammatory enzymes nicotinamide adenine dinucleotide phosphate (NADPH) oxidase and inducible nitric oxide synthase (iNOS) were believed to play major roles. Mounting evidence suggests that activation of NADPH oxidase and the expression of iNOS are directly linked to the generation of highly reactive ROS which affects various cellular components and preferentially damage midbrain dopaminergic neurons in PD. Therefore, appropriate management or inhibition of ROS generated by these enzymes may represent a therapeutic target to reduce neuronal degeneration seen in PD. Here, we have summarized recently developed agents and patents claimed as inhibitors of NADPH oxidase and iNOS enzymes in experimental models of PD.

## 1. Introduction

Brain inflammation may contribute to a wide variety of neurodegenerative pathologies. Major regulators of brain inflammation that may exert a direct effect on neurons are reactive oxygen species (ROSs). ROSs are emerging as important players in the etiology of neurodegenerative disorders [1]. Due to the reduced capacity of neuronal regeneration and high metabolic rate, the brain is believed to be profoundly liable to the damaging effects of ROS and the dopaminergic neurons in the substantia nigra of Parkinson' disease (PD) patients are undoubtedly susceptible. Different data sets suggest that oxidative stress is at the center of various neurodegenerative diseases. Postmortem brain tissues from patients with neurodegenerative diseases including Alzheimer's disease (AD), PD, Huntington's disease (HD), and amyotrophic lateral sclerosis (ALS) clearly show increased levels of ROS in affected brain regions [2–4]. Though one cannot assume that only ROSs are the major cause of these disease states, it is necessary to question what is responsible for this increased ROS generation.

ROSs are a wide range of small signaling molecules which are highly reactive unpaired valence electrons. ROSs include superoxide (O_2_^−^), hydrogen peroxide (H_2_O_2_), hydroxyl radical (OH^•^), and peroxynitrite (ONOO^−^) [5]. Although ROSs have some biological advantages, excessive generation may lead to threatened homeostasis of the biological system [6–9]. ROSs are constantly generated through a variety of pathways, including both enzyme-catalyzed reactions and nonenzyme reactions [10]. Whenever the balance between ROS generation and the natural antioxidant defense system is lost, a series of events may occur deregulating cellular functions which may lead to various pathological conditions for almost all vital organs [11]. ROS can react with vital cell components and alter intrinsic membrane properties like fluidity, ion transport, loss of enzyme activity, protein cross-linking, inhibition of protein synthesis, and DNA damage ultimately resulting in cell death [12]. 

A more direct effect on neurons is the ROS produced by the activation of the several inflammatory enzymes such as nicotinamide adenine dinucleotide phosphate (NADPH) oxidase, the expression of the inducible nitric oxide synthase (iNOS), myeloperoxidase, lipoxygenase, and cyclooxygenase (COX). These enzymes contribute to the pathogenesis of various neurodegenerative diseases including PD. Earlier studies postulated that NADPH oxidase and iNOS are not expressed in normal CNS conditions, but in PD patients and in 1-methyl-4-phenyl-1, 2, 3, 6-tetrahydropyridine (MPTP) intoxicated mice. Both are clearly expressed and activated in glial cells in the ventral midbrain. These two major inflammatory enzymes that produce ROS may have a pathogenic role in PD, because when they are lacking in mutant mice, they show less loss of dopaminergic neurons [13–15].

In this paper, we discuss recent inhibitors of ROS-generating inflammatory oxidative enzymes, in particular the NADPH oxidase and iNOS as a therapeutic strategy for the treatment of PD. We also summarize claims by recent patents for several agents as potential NADPH oxidase and iNOS inhibitors.

## 2. Reactive Oxygen Species and PD

PD, the second most major neurodegenerative disease after AD, is a pathological condition characterized by the degeneration of dopaminergic neurons in the substantia nigra pars compacta and loss of striatal dopamine content [16, 17]. Although several pathogenic hypotheses have been proposed for PD, oxidative stress via the generation of ROS is considered one of the major contributors. Evidence for the role of ROS was first observed in human PD brains showing mitochondrial dysfunction and oxidative damage in the degenerating areas including the substantia nigra [18, 19]. Dopamine is a relatively unstable molecule subject to hydroxyl radical attack that can induce ROS damage both from within and outside the cell [20]. ROS generated from mitochondrial and/or extramitochondrial sources appear to be the main contributor of oxidative stress-mediated neurodegeneration in PD models [16, 21–27]. Importantly, generation of ROS from toxicity induced by accumulation of MPP^+^ in the inner mitochondrial membrane, disruption of complex I in the electron transport chain, and interaction of MPP^+^ with iron stores within the pigmented substantia nigra cells are the known sources of oxidative stress [28–31].

Increasing evidence indicates that inflammatory-activated microglia and astrocytes are considered to be a consequence of neuronal cell death in AD, PD, HD ALS, and multiple sclerosis [32–36]. Activated microglia highly expresses several enzymes including iNOS, NADPH oxidase, COX-2, and myeloperoxidase responsible for inflammatory processes mediated by oxidative stress. These enzymes contribute to the pathogenesis of various neurodegenerative diseases [13, 37–39]. ROS-generating multimeric enzymes are indispensable for protecting the host against infections and injuries. Inappropriate activation of these enzymes may be harmful in noninfectious neurodegenerative disorders [40, 41]. Thus, the discovery of various novel agents that inhibit the activation of these enzymes may prove to be a therapeutic strategy in ameliorating various neurodegenerative diseases including PD.

## 3. Role of NADPH-Oxidase-Derived ROS Signaling in PD

Epidemiological studies suggest that inflammation increases the risk of developing PD [42]. Several studies revealed that a significant source of ROS generated during inflammation was by NADPH oxidase. Nervous system cells contain the NADPH oxidase complex, which, when assembled and activated, produces free radicals in abundance that can lead to tissue damage [43]. NADPH oxidase which is composed of gp91^phox^, p67^phox^, p47^phox^, p40^phox^, and p22^phox^ subunits was first studied because of its essential role in host defense [44]. In resting brain cells, NADPH oxidase is inactive because p47^phox^, p67^phox^, and p40^phox^, which are present in the cytosol as a complex, are separated from gp91^phox^ and p22^phox^, which are transmembrane proteins. Upon activation, the p47^phox^ subunit gets phosphorylated and translocates to the membrane as a complex to assemble with gp91^phox^ and p22^phox^ to form an active NADPH oxidase capable of reducing oxygen to a superoxide radical (O_2_^−^) to generate microglial and/or extramitochondrial-derived ROS [45–48].

Brain regions that are rich in catecholamines, such as adrenaline, noradrenaline, and dopamine, are also exceptionally vulnerable to free radical generation. Catecholamines can spontaneously break down to free radicals or be metabolized to free radicals by endogenous enzymes such as monoamine oxidases. Activated microglia also contribute to the degeneration of dopaminergic neurons by releasing neurotoxic factors such as NADPH-oxidase-derived superoxide and cytokines [49]. Activated microglia can produce a host of toxic molecules including reactive nitrogen species and ROS. Microglia in the vicinity of dopaminergic neurons in disease appears to have an upregulated capacity for ROS production due to increased expression of NADPH oxidase. Release of aggregated and nitrated *α*-synuclein from dying or damaged dopaminergic neurons in the SN is thought to contribute, in part, to their activation [50, 51].

Additionally, neurons in the vicinity of activated microglia may thus be exposed to NADPH-oxidase-derived O_2_^−^ and other secondary oxidants, such as H_2_O_2_. NADPH oxidase can be quickly activated to elevate the level of ROS within a few minutes after stimulation by a variety of growth factors, such as cytokines and hormones including interleukin (IL)-1 [52], platelet-derived growth factor (PDGF) [53], or nerve growth factor (NGF) [54].

Several studies indicated that NADPH oxidase has been linked to microglia-derived oxidative stress from a variety of neurotoxic insults, such as rotenone [55], diesel exhaust particles [56], *α*-synuclein [50], amyloid beta [57], paraquat [58], dopamine neuronal injury [13, 59], and cerebral ischemia-reperfusion injury [60], indicating that microglial NADPH oxidase activation may also be a common denominator of microglial activation associated with neurotoxicity.

## 4. NADPH Oxidase Inhibitors and Experimental Models of PD

It is well documented that NADPH oxidase is upregulated in PD [13]. Reports reveal that NADPH-oxidase activation plays a critical role in the degeneration of dopaminergic neurons and inactivation of this enzyme may be a promising target for PD treatment [13, 55, 61]. Reports also suggest that microglial toxin, LPS-induced loss of nigral dopaminergic neurons *in vivo* and *in vitro*, was significantly less pronounced in NADPH-oxidase-deficient mice, when compared to normal control mice [62]. In the normal CNS, NADPH oxidase is quiescent, but, in patients with PD and in MPTP-intoxicated mice, NADPH oxidase is clearly expressed and activated in glial cells in the ventral midbrain. Thus, agents that inhibit NADPH oxidase activation may be ideal therapeutic agents for the management of PD. Here, we summarize a list of recently published and patented compounds which act as NADPH-oxidase-derived ROS inhibitors in experimental animal models focusing on PD pathology. 

**Figure 1:**
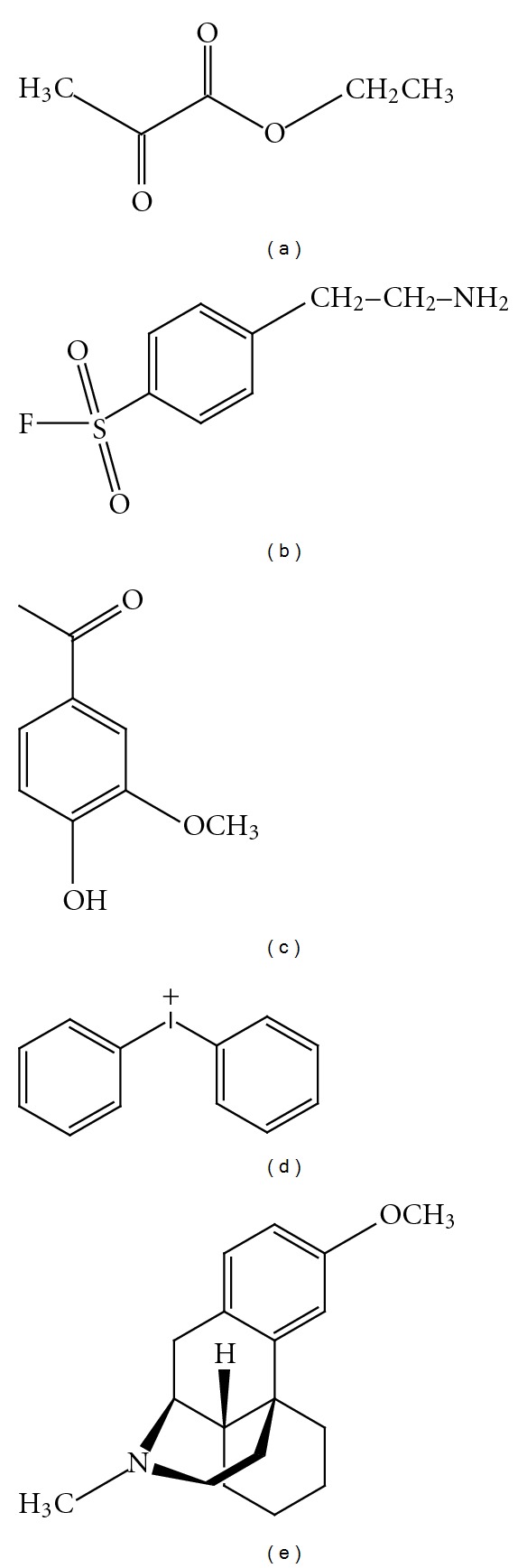
Chemical structure of ethyl pyruvate (a), aminoethyl-benzenesulfonylfluoride (b), apocynin (c), diphenyliodonium (d) and dextromethorphan (e).

Ethyl pyruvate (EP, Figure 1(a)) is an effective scavenger of ROS, especially hydrogen peroxide, by virtue of its nonenzymatic oxidative decarboxylation reaction. EP has been reported to exert pharmacological effects, such as scavenging of ROS, suppression of inflammation, inhibition of apoptosis, and support of cellular ATP synthesis. Recently, EP has been shown to rescue nigrostriatal dopaminergic neurons by regulating glial activation in an MPTP intoxicated mouse model of PD. A single injection of EP (10, 25, and 50 mg/kg body weight) per day administered to mice into the peritoneum at 12 h after the last MPTP injection exerted neuroprotection which was associated with suppression of NADPH-oxidase-derived ROS production by activated microglia [63].

Aminoethyl-benzenesulfonylfluoride (AEBSF, Figure 1(b)), an NADPH oxidase inhibitor, was reported to be useful in ameliorating oxidative stress and apoptosis in mesencephalic dopaminergic neuronal cells. AEBSF at 300 *μ*M significantly blocked 1-methyl-4-phenylpyridinium ion-(MPP^+^-) induced ROS production for over 45 min in N27 cells in a dose-dependent manner and rescued the cells from apoptotic cell death. The study supported that NADPH oxidase play a critical role in the oxidative damage in PD and inhibiting this enzyme activation by AEBSF may lead to novel therapy for PD [61].

Apocynin (4-hydroxy-3-methoxyacetophenone, Figure 1(c)), a selective inhibitor of NADPH-oxidase, can block the production of superoxide and oxygen-free radicals that typically accompany inflammation. Apocynin is a compound originally isolated from the medicinal plant *Picrorhiza kurroa*, which inhibited both intracellular and extracellular ROS production by interfering with the phagosomal association of the cytosolic protein p47^phox^. Apocynin has been used to prevent oxidative-stress-mediated cell damage in several disease models. Recently, apocynin has been proved to improve neurological function and can be used as a neuroprotective agent [64–68]. In the presence of microglial NADPH oxidase activation *in vitro*, apocynin was able to reduce extracellular ROS and cellular damage. Apocynin pretreatment (0.5 mM for 30 min) also attenuated the rotenone-induced release of superoxides from activated microglia mediated by NADPH oxidase [55].

In another study, exposure to paraquat (50 *μ*M), a herbicide with a structure similar to the neurotoxin MPP^+^, has been shown to produce PD-like symptoms and generated ROS (including superoxide anions) in BV-2 cells. Paraquat-induced cytotoxicity in BV-2 cells was accompanied by translocation of the p67^phox^ cytosolic subunit of NADPH oxidase to the membrane. Paraquat-induced ROS production was significantly inhibited by apocynin (1 mM) [69]. Data from the studies above indicated that apocynin may inhibit the release of microglial NADPH-oxidase-mediated superoxide in microglia-enhanced degeneration of dopaminergic neurons.

 Diphenyliodonium (DPI, Figure 1(d)) is a widely used selective NADPH oxidase inhibitor which was documented to act as a neuroprotective agent due to its potent anti-inflammatory properties mediated through the inhibition of ROS. At very low concentrations, DPI inhibited both extracellular superoxide and intracellular ROS production in dopaminergic neurons from LPS-induced degeneration and in LPS-treated microglial activation [70]. In a report by Ma and Zhou, DPI at 3–30 *μ*M concentrations inhibited intrinsic NADPH oxidase activity in N27 cells. Furthermore, DPI blocked MPP^+^-induced ROS production. DPI also promoted the survival of primary striatal neurons [71] and protected against glutamate-induced apoptosis in dopaminergic SH-SY5Y cells [72]. In addition, several *in vivo* studies demonstrated that DPI delivered protection against global cerebral ischemia [64], rotenone- [55], paraquat- [73], 6-OHDA- [74], MPTP- [59], and IFN-*γ*/LPS- [75] induced dopaminergic degeneration. In a recent study, Gao et al. [76] developed a two-hit (neuroinflammation and mutant *α*-synuclein (*α*-syn) overexpression) animal model to investigate mechanisms through which mutant *α*-syn and inflammation work in concert to mediate chronic PD neurodegeneration. Results revealed that LPS stimulation within the brain to transgenic mice over expressing human A53T mutant *α*-syn developed persistent neuroinflammation, chronic progressive degeneration of the nigrostriatal dopamine pathway, accumulation of aggregated, nitrated *α*-syn, and formation of Lewy body-like inclusions in nigral neurons. Continuous inhibition of NADPH oxidase by 4-week infusion of DPI (5 *μ*g/kg/h) blocked *α*-syn pathology and nigral neurodegeneration. These studies provide a potential role of DPI in the treatment of PD.

 Dextromethorphan (DM, Figure 1(e)) is neuroprotective through inhibition of microglial activation and NADPH oxidase activation. Earlier reports reveal that DM protects dopaminergic neurons against inflammation-mediated degeneration induced by LPS, through inhibition of superoxide radicals [77]. Furthermore, Li et al. [78] reported that femto- (10^−13^ and 10^−14 ^M) and micromolar (10^−5^ to 10^−7 ^M) concentrations of DM (both pre- and posttreatment) showed equal efficacy in protecting LPS-induced dopaminergic neuron death in midbrain neuron-glia cultures. These studies indicated that the neuroprotective effect elicited by femtomolar concentrations of DM is mediated through the inhibition of LPS-induced proinflammatory mediators, especially superoxide. In another study, DM was reported to elicit neuroprotective effects in the MPTP-intoxicated PD model in mice. DM significantly reduced the MPTP-induced production of both extracellular superoxide free radicals and intracellular ROS in both *in vitro* and *in vivo* experiments [79].

**Figure 2:**
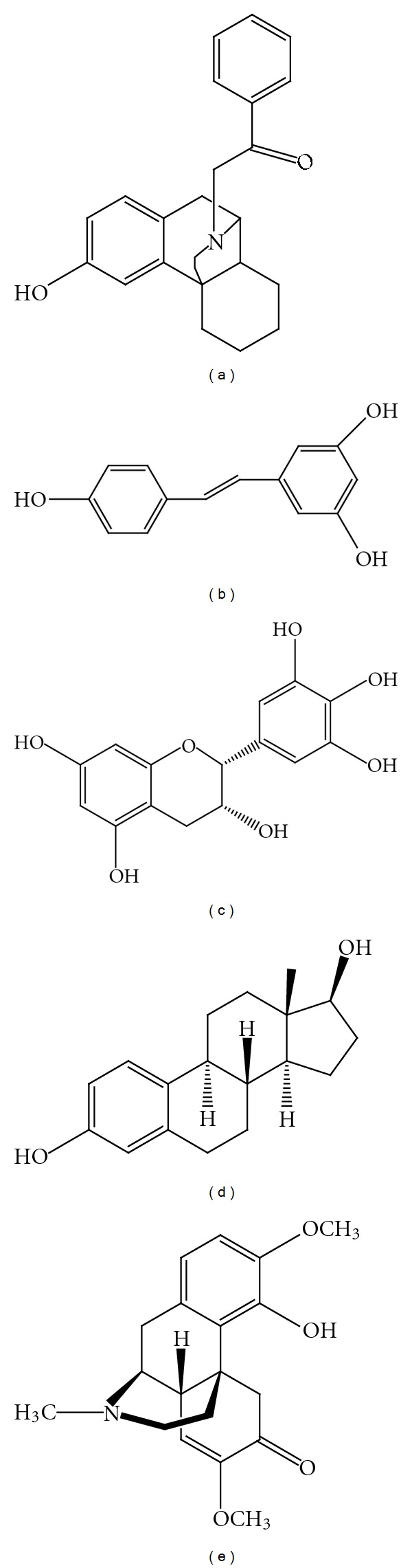
Chemical structure of 3-hydroxymorphinan (a), resveratrol (b), epigallocatechin (c), estradiol 17-*β* (d), and sinomenine (e).

Recently, Ramanathan et al. [80] revealed that DM increased superoxide dismutase (SOD) and catalase, reduced thiobarbituric acid reactive substances in the hippocampal and striatal regions of monosodium glutamate-induced neurodegeneration in rats, and improved neuroprotection based on its antioxidant properties. Another recent report also indicated that increased ROS production in activated BV-2 microglial cells by LPS was associated with increased expression of NADPH oxidase (NOX)-2, a subunit component of NADPH oxidase and DM significantly suppressed the upregulation of NOX-2 as well as subsequent ROS production in activated BV-2 cells [81]. In light of this, DM may form the potential therapeutic strategy for the treatment of PD. The metabolite of DM, 3-hydroxymorphinan (Figure 2(a)), also protected the nigrostriatal pathway against MPTP-elicited damage both *in vivo* and *in vitro* by reducing MPTP-elicited reactive microgliosis as evidenced by the decreased production of ROS due to its potent neuroprotection in PD [82].

 Resveratrol (3, 4, 5-trihydroxy-*trans*-stilbene, Figure 2(b)), a nonflavonoid polyphenol naturally found in red wine and grapes, has been found to possess antioxidant, anticancer, and anti-inflammatory properties. Recent results from Zhang et al. [83] clearly demonstrated that resveratrol pretreatment (15–60 *μ*M) for 30 min stimulated with LPS (10 ng/mL) protected dopaminergic neurons against LPS-induced neurotoxicity in concentration and time-dependent manners through the inhibition of microglial activation and the subsequent reduction in release of proinflammatory factors. The authors showed that resveratrol reduced NADPH-oxidase-mediated generation of ROS and inhibited the LPS-induced translocation of NADPH oxidase cytosolic subunit p47^phox^ to the cell membrane. The most important finding is that resveratrol failed to exhibit neuroprotection in cultures from NADPH-oxidase-deficient mice. This data indicates that NADPH oxidase may be a major player in resveratrol-mediated neuroprotection in the models of PD.

The epigallocatechin (EGCG, Figure 2(c)), a catechin polyphenol, was reported to reduce neuronal NADPH expression in rats exposed to acute hypoxia [84]. EGCG was also reported to inhibit cytosolic subunits of NADPH oxidase from translocating into the membrane, suggesting that inhibition of NADPH oxidase activity may prevent oxidative stress. In a recent report, the effects of EGCG on dichlorodiphenyltrichloroethane (DDT), a pesticide which is believed to play a causative role in the etiology of PD, was studied. It was found that EGCG concentration dependently (1 *μ*M, 3 *μ*M, and 10 *μ*M) reduced DDT-induced cell death in dopaminergic SH-SY5Y cells. Reports also indicated that EGCG was capable of reducing dopaminergic neurotoxin 6-hydroxydopamine-(6-OHDA-) induced cell death in SH-SY5Y cells [85] and MPTP-induced neurotoxicity in mice [86]. The authors suggest that consumption of green tea, which contains high concentrations of EGCG, may provide potential prophylactic effects in reducing the risk of developing PD.

Few neuropeptides such as pituitary adenylate cyclase-activating polypeptide (PACAP) 38, PACAP 27, and its internal peptide, Gly-Ile-Phe (GIF), were reported to be neuroprotective at 10^−13 ^M against LPS-induced dopaminergic neurotoxicity. PACAP is widely distributed in the peripheral and central nervous system, where PACAP release is reported to serve as a neuronal survival factor [87, 88]. PACAP is reported to have diverse functions and has been shown to act as a neurotransmitter/neuromodulator [89] and a neuroprotectant [90, 91]. PACAP 38 and GIF also protected against MPTP-induced neurotoxicity in animal models. The polypeptides significantly ameliorated the production of microglia-derived ROS, where both LPS- and phorbol 12-myristate 13-acetate-induced superoxide and intracellular ROS were inhibited. The study showed that PACAP38 and GIF were neuroprotective only in normal cultures and not in NADPH oxidase deficient cultures, proving the important role of NADPH oxidase for GIF and PACAP 38s neuroprotection [92].

The steroid hormone, 17*β*-estradiol (E2, Figure 2(d)), is released into the blood where it can exert trophic or regulatory effects on many different target tissues such as the breast, ovary, uterus, bone, and brain. Reports revealed that E2 treatment (0.025 mg; 14–21 day release via minipumps) strongly attenuated the elevation of NADPH oxidase activity in the hippocampal CA1 region following cerebral ischemia in brain, which correlated with its suppression of O_2_^−^ levels and its neuroprotective effect [93]. Moreover, E2 inhibited activation of the GTPase, Rac1, in an Akt-dependent manner following cerebral ischemia, which is critical for NOX-2 activation. Due to its neuroprotective effect and potent role in inhibiting NADPH oxidase expression, E2 may be further developed for the treatment of PD [94].

 Transforming growth factor (TGF)-*β*1 is a pleiotropic cytokine that plays a critical role in the control of cell growth, differentiation, inflammation, cell chemotaxis, apoptosis, and hematopoiesis. Studies have shown that TGF-*β*1 can protect neurons from cell death induced by oxidative injury [95]. A recent report by Qian and Flood [96] revealed that the neuroprotective effects of TGF-*β*1 are mainly attributed to its ability to inhibit the production of ROS from microglia during their activation or reactivation. TGF-*β*1 inhibited LPS-induced NADPH oxidase subunit p47^phox^ translocation from the cytosol to the membrane in microglia, thereby exerting potent anti-inflammatory and neuroprotective properties.

Sinomenine (Figure 2(e)), a natural dextrorotatory morphinan analog, was reported to possess anti-inflammatory and neuroprotective properties by the inhibition of microglial NADPH oxidase. Sinomenine pretreatment for 30 min at micromolar (10^−6^–10^−5 ^M) and subpicomolar concentrations (10^−14^–10^−13 ^M) showed equivalent efficacy in protecting against dopaminergic neuron death in rat midbrain neuron-glial cultures. Furthermore, sinomenine suppressed LPS-induced extracellular ROS production via the inhibition of NADPH cytosolic subunit p47^phox^ translocation to the cell membrane. These findings strongly suggest that the protective effects of sinomenine are most likely mediated through the inhibition of microglial NADPH oxidase activity [70].

**Figure 3:**
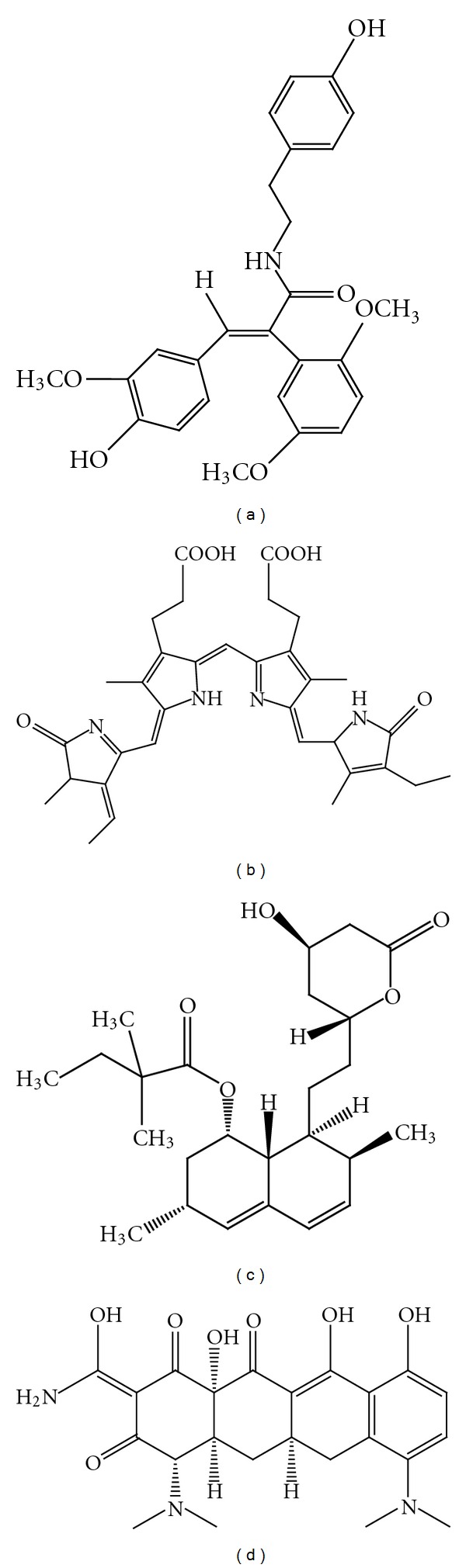
Chemical structure of FLZ (a), phycocyanobilin (b), simvastatin (c), and minocycline (d).

N-[2-(4-hydroxy-phenyl)-ethyl]-2-(2,5-dimethoxy-phenyl)-3-(3-methoxy-4-hydroxy-phenyl)-acrylamide (FLZ, Figure 3(a)) is a squamosamide derivative reported to mediate anti-inflammatory and neuroprotective effects in both LPS and MPTP-intoxicated models of PD [97]. For *in vivo* studies, FLZ (75 mg/kg, p.o.) was administered 30 min before every MPTP injection (15 mg/kg, s.c.) for 6 consecutive days. For LPS (2 ng/mL) stimulation, 10 *μ*M of FLZ was pretreated for 1 h. The neuroprotective effect of FLZ was attributed to a reduction in LPS-induced microglial production of proinflammatory factors such as superoxide, tumor necrosis factor-*α* (TNF-*α*), nitric oxide (NO), and prostaglandin E2 (PGE_2_). Findings from this study revealed that the anti-inflammatory properties of FLZ were mediated through inhibition of NADPH oxidase, the key microglial superoxide-producing enzyme [97].

Phycocyanobilin (PCB, Figure 3(b)), a chromophore derived from biliverdin, plays an essential light-harvesting role in many blue-green algae and cyanobacteria. It constitutes up to 1% of the dry weight of spirulina. Recently, it was reported that C-phycocyanin administered orally (the spirulina holoprotein that includes PCB) suppresses the neurotoxic impact of the excitotoxin kainite in rats, and a diet high in spirulina ameliorates the loss of dopaminergic neurons in the MPTP-induced Parkinsonian syndrome in mice. The central physiological effects of PCB may also reflect inhibition of neuronal NADPH oxidase, which is known to have a modulatory impact on neuron function, and can mediate neurotoxicity in certain neurodegenerative diseases [98]. PCB has been shown to be a potent inhibitor of NADPH oxidase activity in various human cell cultures at micromolar concentrations. PCB may thus have versatile potential for preserving the healthy function of the CNS in advanced old age patients suffering from neuroinflammatory diseases including PD.

 In a recent study, Santiago et al. [99] investigated the effect of simvastatin (Figure 3(c)) a commonly used, cholesterol-lowering drug, in LPS and MPTP neurodegenerative models to identify its neuroprotective effects for PD. The study suggested that simvastatin (5 mg/kg body weight, i.p.) could prevent neurotoxic damage by LPS stimulation in microglial cells. Studies by Brenneman et al. [90] also indicated that simvastatin is associated with a reduced incidence of dementia and PD in elderly patients. Simvastatin treatment (10 *μ*M) blocked the rac1-dependent activation of NADPH oxidase and O_2_^−^ production and significantly diminished microglial CC chemokine ligand 5 (a major chemo attractant of inflammatory cells) expression induced by interferon-*β* alone or by a combination of interferon-*β*/TNF-*α*, thereby exerting its suppressive effects on inflammation in the CNS [100]. Furthermore, simvastatin inhibited NADPH oxidase and the production of ROS in microglia [101]. A recent study showed that simvastatin protects dopaminergic neurodegeneration in *in vivo* parkinsonian models [102]. Further, simvastatin was also reported to attenuate superoxide generation by NADPH oxidase activation, protecting the endothelial cell barrier [103]. All these data suggest the protective effect of simvastatin against the degeneration of dopaminergic neurons and may be developed as a promising drug to provide neuroprotection in PD.

Minocycline (Figure 3(d)), a well-known semisynthetic tetracycline derivative, is neuroprotective in several animal models of neurodegeneration, including PD [104, 105]. Studies have demonstrated that the neuroprotective actions of minocycline are attributable to inhibition of microglial activation accompanied by oxidative stress. Choi et al. [106] reported that minocycline (25 or 50 mg/kg) exerted neuroprotection by significantly attenuating thrombin-induced neurotoxic effects through inhibition of NADPH oxidase activation and ROS production from activated microglia.

Several patents for various categories of compounds have been claimed for selectively inhibiting NADPH oxidase by proving to be useful in the treatment and/or prevention of inflammatory conditions in neurodegenerative diseases. Patented compounds published over the last five years for selectively inhibiting NADPH oxidase were collectively described in our earlier review [107]. The most recent relevant patents showing a possible role in ameliorating neurodegenerative diseases such as PD include the pyrazolo pyridine derivatives [108], tetrahydroindole derivatives [109], imipramine blue analogs [110], quinolone derivatives [111], and hesperidin and hesperetin analogs [112]. These compounds were shown to selectively inhibit and downregulate the expression of NADPH oxidase by suppressing ROS generation, consequently proving their importance as novel therapies in ameliorating neuroinflammatory degenerative diseases including PD.

## 5. Role of iNOS-Derived ROS Signaling in PD

Apart from the above-discussed NADPH-oxidase-derived ROS systems, focus also points to nitrogen dioxide-derived reactive species such as ONOO^−^, NO, and other unrecognized potential reactive nitrogen species as the main culprits [113]. Due to their highly unstable nature and reactivity, biological molecules such as proteins, DNA, and lipids in dopaminergic neurons in the brains of parkinsonian patients could be targeted for oxidation resulting in extensive cellular injury and cell death. Normally, inducible NO synthase (iNOS) is not expressed in the brain, but in pathological situations, especially those associated with gliosis, iNOS can be induced.

In experimental PD models and MPTP neurotoxin-induced models, induction of iNOS expression has been observed [14, 114]. Earlier studies have also suggested that inhibition of iNOS showed neuroprotection in the MPTP-induced PD model. In addition, inflammatory mediators such as LPS and cytokines also cause an increase in iNOS expression in microglia and astrocytes [115] and possibly in neurons [116]. Once expressed, iNOS produces high levels of NO continuously from microglia or astrocytes [117, 118].

Nitric oxide, a lipophilic diatomic molecule, can travel several micrometers away from its site of production and freely cross the plasma membrane to reach the intracellular space of dopaminergic neurons. Also, the interaction of NO and O_2_^−^ will result in the formation of OONO^−^, a highly reactive species. Peroxynitrite is a potent cytotoxic oxidant, which in turn will inflict oxidative damage to biological targets such as inactivating ion channels, damaging DNA, and nitrating tyrosine residues that can potentially inactivate enzymes and disrupt signal transduction [119]. Therefore, inhibition of glial activation-mediated oxidative stress by reducing the iNOS may have therapeutic value in the treatment of neuroinflammation related to PD. In the following section, we describe recently available agents and patented compounds that selectively inhibit iNOS activity and may show a promising role in PD treatment.

## 6. Inducible Nitric Oxide Synthase Inhibitors and Experimental Models of PD

**Figure 4:**
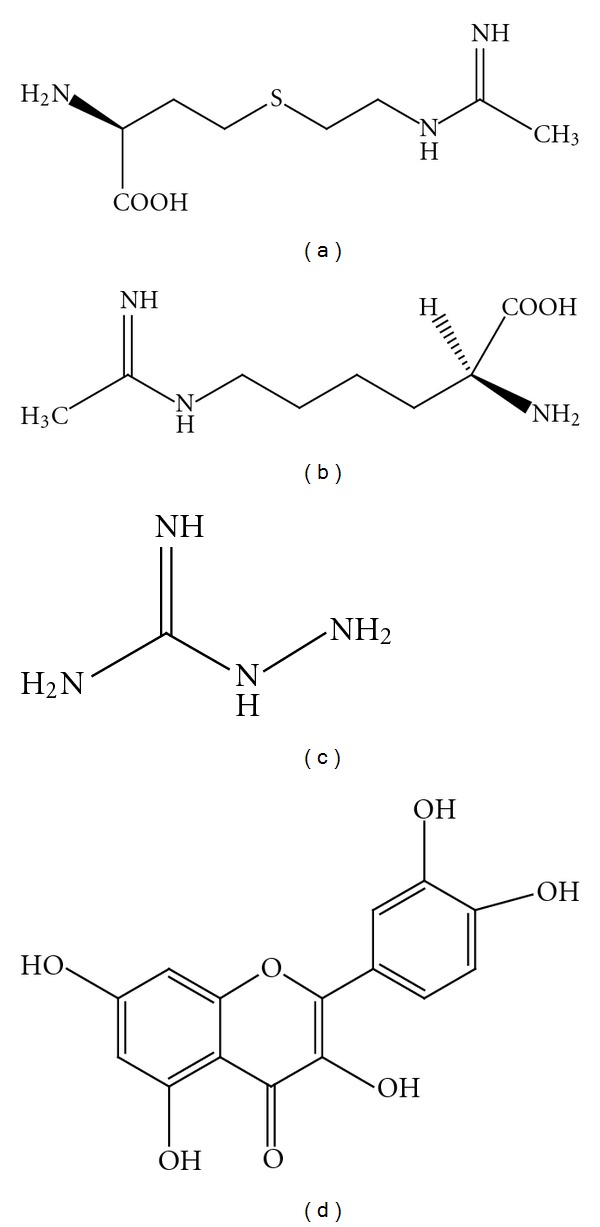
Chemical structure of GW274150 (a), L-NIL (b), aminoguanidine (c), and quercetin (d).

It is well documented that experimental PD models using various toxins and MPTP-induced dopaminergic degeneration express high levels of iNOS [37, 120]. Recently, Broom et al. [121] reported a selective iNOS inhibitor, GW274150 ([2-[(1-iminoethyl) amino] ethyl]-L-homocysteine, Figure 4(a)) showing a potent role in the pathogenesis of PD. They indicated that 6-OHDA administration produced an increased number of cells expressing iNOS and was also associated with increased microglial activation. GW274150 treatment (3, 10, and 30 mg/kg) orally twice daily for 7 consecutive days leads to a suppression of iNOS expression and the inflammatory response in the 6-OHDA model of PD and was neuroprotective but at a narrow therapeutic range. The findings from this paper support the concept that NO has a detrimental effect on dopaminergic neurons and that modulation of the inflammatory response may be a valid neuroprotective therapeutic approach in treating PD.

 Gahm et al. [122] reported that L-*N*-iminoethyl-lysine (L-NIL, Figure 4(b)), a selective iNOS inhibitor, appeared to protect the injured brain by limiting OONO^−^ formation. Their study analyzed a variety of parameters including neuronal degeneration, survival, cellular apoptosis, and formation of nitrotyrosine following traumatic brain injury (TBI). L-NIL significantly reduced iNOS activity in animals injured by brain contusion. Moreover, neuronal degeneration and NT immunoreactivity significantly reduced at 24 h. Neuronal survival was unchanged at 24 h but increased at 6 days in L-NIL-treated animals. Cellular apoptosis of mononuclear phagocytes (ED-1) and neuron (NeuN) positive cells were significantly reduced following L-NIL treatment 6 days after trauma. These findings also strongly support a putative harmful role of iNOS induction early after TBI and the neuroprotective role of L-NIL in neurodegenerative diseases such as PD.

 The neuroprotective actions of aminoguanidine (AG, Figure 4(c)) have long been studied. Several authors reported that the selective inhibition of iNOS is one of the major mechanisms by which AG exerts its neuroprotection [123–125]. Cash et al. [126] reported the neuroprotective effects of AG on transient focal ischemia in the rat brain. Lu et al. [127] proposed the neuroprotective action of AG by combined magnetic resonance imaging and histopathologic and functional analysis after lateral fluid-percussive brain injury in rats. The cerebroprotective effect of AG in a rodent model of stroke was also studied [128]. In a recent report [129], the effectiveness of AG was studied in modulating the toxicity of aluminum chloride on the nitrite levels, malondialdehyde concentration, reduced glutathione content, and cytochrome c oxidase activity in Wistar rats and confirmed that the inhibition of iNOS was responsible for this action. All these studies have concluded that the potent inhibition of iNOS activity was partly responsible for neuroprotection and AG can be further developed in the prevention of various CNS disorders including PD.

 Quercetin (Figure 4(d)), a major flavonoid naturally occurring in plants, deserves attention because of its beneficial effects observed in various *in vitro* and *in vivo* neural damage models. Quercetin significantly exerted a neuroprotective effect through inhibition of the iNOS/NO system and proinflammation gene expression in PC12 cells and in Zebrafish [130]. The selective dopaminergic neurotoxin 6-OHDA was used to induce neural damage in PC12 cells and Zebrafish. Pretreatment with quercetin offered neuroprotection against 6-OHDA-induced PC12 apoptotic cell death and dopaminergic neuronal loss in Zebrafish. A mechanistic study revealed that quercetin could inhibit NO overproduction and iNOS overexpression in PC12 cells and downregulates the overexpression of proinflammatory genes suggesting that role of quercetin in neuroprotection leading to its development as an effective therapeutic agent for the treatment neurodegenerative diseases including PD.

**Figure 5:**
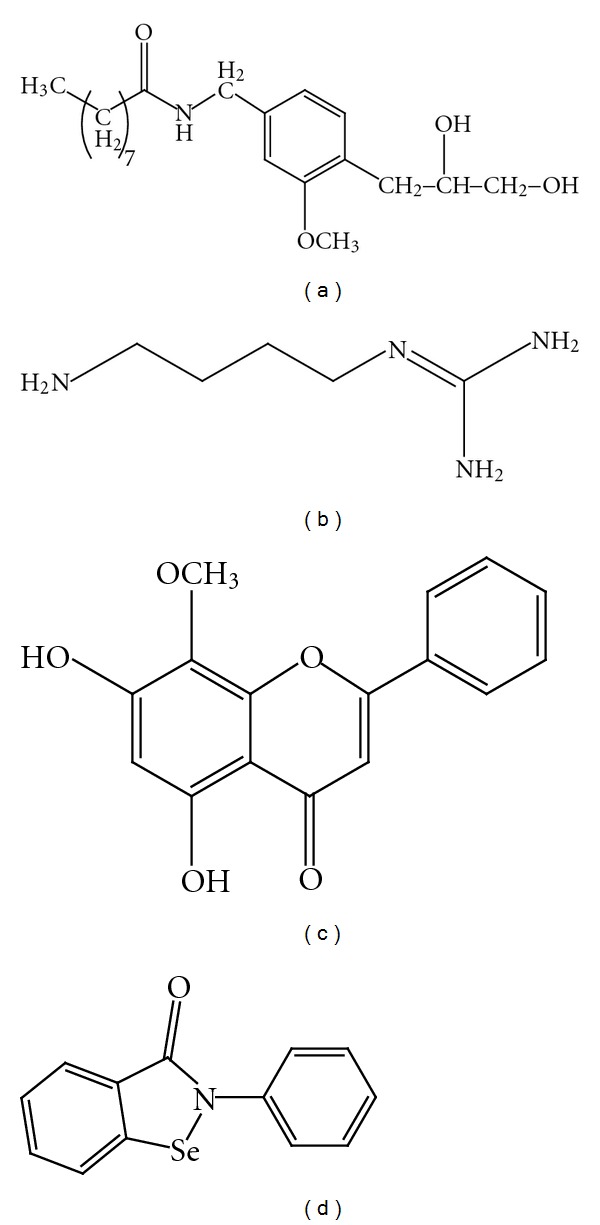
Chemical structure of glyceryl nonivamide (a), agmatine (b), wogonin (c), and ebselen (d).

 The neuroprotective effects of glyceryl nonivamide (GLNVA, Figure 5(a)), a vanilloid receptor (VR) agonist on activated microglia and 6-OHDA-induced neurotoxicity in dopaminergic SH-SY5Y cells were studied recently [131]. The authors revealed that GLNVA decreased LPS-activated microglia-induced overexpression of neuronal nitric-oxide synthase and gp91^phox^ on SH-SY5Y cells. GLNVA (1, 10, 100 *μ*M) for 24 h diminished LPS-induced NO production, overexpression of iNOS, and intracellular reactive oxygen species in activated microglia. 6-OHDA-induced overexpression of nNOS, iNOS, COX-2, and gp91^phox^ were also reduced by GLNVA in SH-SY5Y cells. The neuroprotective effects of GLNVA are mediated, at least in part, by decreasing the inflammation- and oxidative-stress-associated factors induced by microglia and 6-OHDA.

 The neuroprotective effects of exogenous agmatine, a guanidinium compound (Figure 5(b)), were investigated in experimental spinal cord injury (SCI). Agmatine is a neurotransmitter—neuromodulator with both *N*-methyl-d-aspartate receptor (NMDAR)—antagonizing and NO synthase-inhibiting activities. Agmatine administration following SCI was shown to reduce NO levels significantly and suggested that this drug may be helpful in the treatment of patients with neurodegeneration especially in SCIs [132]. In a recent study, the effects of agmatine on cell injury induced by rotenone commonly used in establishing *in vivo* and *in vitro* models of PD in a human-derived dopaminergic SH-SY5Y cell line were shown. Agmatine dose-dependently suppressed rotenone-induced cellular injury through a reduction of oxidative stress, by protecting dopaminergic neurons [133].

 Wogonin (5, 7-dihydroxy-8-methoxyflavone, Figure 5(c)), an active component originated from the root of *Scutellaria baicalensis*, has been reported to possess antioxidant and anti-inflammatory properties. Wogonin (5, 20, 50 mM) inhibited inflammatory activation of cultured brain microglia by diminishing LPS-induced NO production via suppressing iNOS induction in microglia [134]. Furthermore, Chun et al. [135] reported the inhibitory activities of wogonin derivatives on LPS-induced NO production in BV-2 microglial cells and on H_2_O_2_-induced neuronal cell death in SH-SY5Y cells. Wogonin and its derivatives ranging between 5, 10, 20, and 40 *μ*M concentration decreased the production of NO and inflammatory cytokines owing to their potential role in mitigating neuroinflammation seen in PD.

 2-Phenyl-1,2-benzisoselenazole-3(2H)-one (ebselen), a seleno organic compound and a strong ONOO^−^ cleansing agent (Figure 5(d)), possesses antioxidant and anti-inflammatory properties and is now under clinical trials for the treatment of ischemic stroke. Earlier reports indicated that ebselen can also preferentially inhibit the activity of inducible nitric oxide (NO) synthase within a certain concentration range [136]. Ebselen prevented both neuronal loss and clinical symptoms in a primate MPTP model of PD. Ebselen (10 *μ*M) also prevented peroxide radical overproduction induced by serum withdrawal in cultured PC12 cells and hydroxyl radical generation induced by the mitochondrial toxin, MPP^+^, in an *in vivo* system in the rat brain [137]. The authors indicated that ebselen inhibited the free radical production and may be useful as preventive treatment in PD.

**Figure 6:**
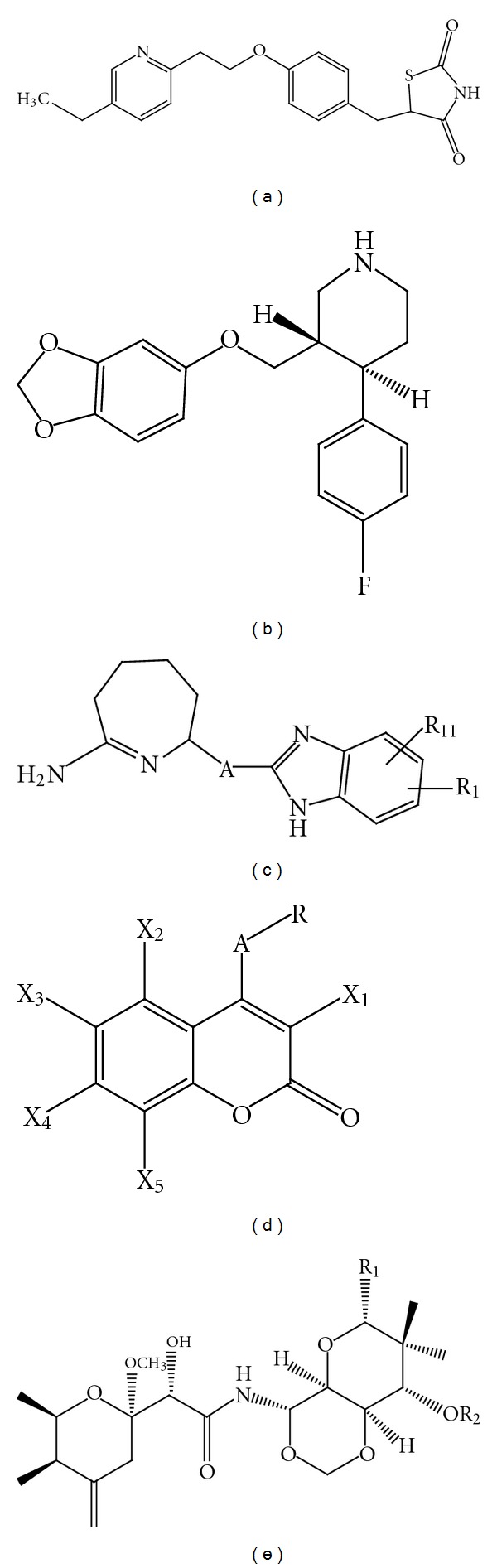
Chemical structure of pioglitazone (a), paroxetine (b), imidazopyridine (c), coumarins (d), and theopederin derivatives (e).

Pioglitazone, a PPARgamma agonist (Figure 6(a)), was reported to protect mice from MPTP-induced dopaminergic cell loss, glial activation, and loss of catecholamines in the striatum. In addition, pioglitazone (10 *μ*M) provided neuroprotective properties to substantia nigra dopaminergic neurons in LPS-induced PD models both *in vivo* and *in vitro* [138, 139]. In mice treated with pioglitazone, there was reduced activation of microglia, reduced induction of iNOS-positive cells and less glial fibrillary acidic protein positive cells in both the striatum and substantia nigra pars compacta. A comprehensive mechanistic study revealed that pioglitazone-mediated neuroprotection involves inhibition of microglial activation and decreased expression and activity of iNOS and may offer a treatment opportunity in PD to slow the progression of disease that is mediated by neuroinflammation [138, 140].

Recently, the antidepressant paroxetine (Figure 6(b)) was reported to promote the survival of nigrostriatal dopaminergic neurons in the MPTP mouse model of PD. Treatment with paroxetine (10 mg/kg body weight, equivalent to 0.2–0.25 mg/day) into the peritoneum for 6 days, beginning at 12 h after last MPTP injection, prevented the degeneration of nigrostriatal DA neurons, increased striatal dopamine levels, and improved motor function. The authors indicated that the neuroprotection afforded by paroxetine may partly be associated with the suppression of iNOS in activated microglia [141].

During the last five years, several novel compounds that selectively inhibit iNOS expression have been patented. Thomas et al. [142] claimed the usefulness of imidazopyridine derivatives (Figure 6(c)) as iNOS inhibitors. They found the novel 7-amino-3, 4, 5, 6, tetrahydro-2H-azepin-2-yl-substituted imidazopyridine derivatives have valuable pharmacological properties which make them commercially utilizable. They are selective inhibitors of the enzyme iNOS. On account of their iNOS-inhibiting properties, the compounds according to the invention can be employed in human, veterinary medicine, and therapeutics, where an excess of NO or O_2_^−^ is involved due to iNOS activation. They can be used without limitation for the treatment and prophylaxis of various neuroinflammatory and neurodegenerative diseases including PD [142].

A patent has also been obtained by Sharon et al. [143] on a few novel coumarins (Figure 6(d)) for their use as inhibitors of iNOS. Their study reveals that the compounds synthesized from their invention can be used for various neurodegenerative diseases including PD. 

Recently, Tadayoshi et al. [144] filed a patent for a sense oligonucleotide with sequence complementary to a single-stranded RNA (antisense transcript) with sequence complementary to mRNA of the iNOS gene in order to control expression of iNOS. The authors reveal that the proposed invention can control the expression of iNOS and can be useful for biological defense in ameliorating diseases related to excessive production of NO, such as neuroinflammation.

Theopederin and derivatives (Figure 6(e)) are marine natural substances isolated and purified from *Porifera* species. In a recent patent, a pharmaceutical and health food preparation containing theopederin derivatives as active constituents was claimed by Heonjoong and Hyun-Sil [145]. The authors claim that these derivatives selectively inhibit the excessive generation of NO and iNOS activation and are useful to treat and/or prevent various diseases including autoimmune diseases, inflammatory diseases, multiple sclerosis, and neurodegeneration.

There has also been a patent filed by Singh [146] on the exemplary compound lovastatin, which is a sodium salt of phenylacetic acid, FPT inhibitor II, N-acetyl cysteine, and cyclic AMP, selectively inhibit the iNOS activation in LPS-stimulated microglial cells, and can be used for the treatment of various neuroinflammatory diseases.

**Figure 7:**
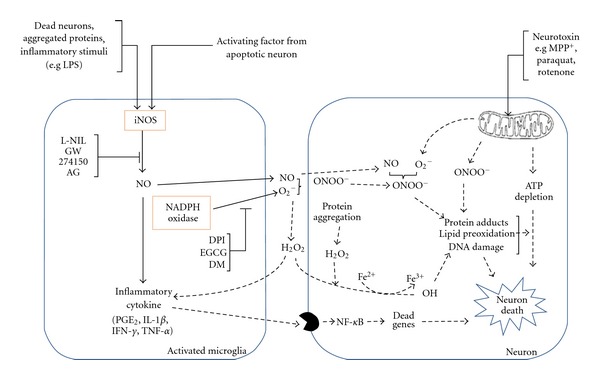
Role of iNOS and NADPH oxidase and their inhibitors in oxidative stress and neuronal damage. Reactive oxygen species (ROS) can be generated in distinct ways, for example, cell activation by neurotoxins (MPTP, paraquat, and rotenone), and mitochondrial dysfunction and protein aggregation. ROSs have a role in oxidative stress, thereby causing neuronal injury or cell death which can be a factor for microglial activation. Several inhibitors of iNOS (L-N-iminoethyl-lysine (L-NIL), GW 274150, and aminoguanidine (AG)) and NADPH oxidase inhibitors diphenyliodonium (DPI), epigallocatechin (EGCG), and dextromethorphan (DM) as shown inhibit the generation of nitric oxide and superoxides, thereby inhibiting the formation of ROS and preventing neurodegeneration. Abbreviations: iNOS: inducible nitric oxide synthase, LPS: lipopolysaccharides, NADPH oxidase: nicotinamide adenine dinucleotide phosphate-oxidase, NO: nitric oxide, O_2_^−^: peroxy radical, ONOO^−^: peroxynitrite, NF-*κ*B: nuclear factor kappa, H_2_O_2_: hydrogen peroxide, TNF-*α*: tumor necrosis factor-alpha, PGE_2_: prostaglandin E2, IL-1*β*: interleukin-1 beta, and IFN-*γ*: interferon-gamma.

The role of iNOS and NADPH oxidase in oxidative stress and neuronal damage and the potential therapeutic strategy of the discussed compounds were represented in Figure 7. In view of the published reports and patents filed, inhibition of iNOS and NADPH oxidase by various existing and emerging molecules would be one of the ideal targets for the treatment for PD.

## 7. Conclusion

The etiology of PD remains unknown, and the mechanisms controlling the selective and progressive degeneration of the nigrostriatal dopaminergic pathway are poorly understood. Therapeutic intervention aimed at halting the degenerative nature observed in PD is of prime importance and presents some research opportunities. Studies of postmortem PD brains and various cellular and animal models of PD in the last two decades strongly suggest that the neuroinflammation caused by oxidative stress is one of the major mechanisms in PD pathology. It is believed that activated glial cells, which comprise the majority of this inflammatory response, contribute to neurodegeneration through the production of ROS. Currently, ROS-generating oxidative enzymes are emerging as major therapeutic targets to inhibit neurotoxicity seen in PD.

A range of data during the past few years suggest that anti-inflammatory agents with neuroprotective effects by inhibiting inflammatory oxidative enzymes in experimental models may be capable of preventing or reducing neuronal degeneration and arrest the progression of PD. Although several anti-inflammatory agents were known to inhibit the ROS, none has been specific and the results achieved are somewhat inconsistent and show limited mechanistic relevance to PD. Thus, inhibitors of major ROS-generating NADPH oxidase and iNOS that have a decent proven record of safety and efficacy would be promising candidates. The compounds discussed in this paper provide valuable information in selectively inhibiting the ROS generated by oxidative enzymes in *in vitro* and *in vivo* experimental models of PD. These compounds must be proved for their safety, selectivity, toxicity, bioavailability, therapeutic window, and absence of significant side effects. Furthermore, combination therapy of selected agents in the correct time frame and dose may also provide better results to achieve synergistic clinical effects.

However, a complete understanding of the molecular mechanisms of the specificities of ROS in PD, and larger studies both epidemiologic and randomized clinical trials in humans, as well as animal studies, are needed to validate these findings in delivering beneficial effects in the treatment of PD.
